# A Case of Anal Fissure

**Published:** 1887-06

**Authors:** E. W. Berridge

**Affiliations:** London


### A CASE OF ANAL FISSURE.

E. W. Berridge, M. D., London.

Dr. Clarence Butler's "happy coincidence" reminds me of my own similar good fortune in curing anal fissure by the *simillimum* without resorting to "forcible dilatation," as advocated by a writer in the *Homœopathic* (?) *Journal of Obstetrics*, April 30th, 1880. Miss A. two years ago had an anal fissure cauterized under Chloroform; since then the stools have been difficult, sometimes large, occurring daily, but with much straining; the stool seems as if it had to force its way through, and sometimes the integument cracks and hemorrhage follows; after stool she is exhausted, and has a feeling of a swelling as large as her fist internally in rectum. She is subject to chilblains. Menses regular; before menses feels depressed, as if she would like to hide herself, tired of life, and would like to commit suicide; this mental state has lasted for four or five years. Fullness after a meal. Used to have a feeling after a stool as if some remained behind, but not lately, as she has been taking Manna. Once *Graphites*$^{3}$ temporarily relieved her. She also consulted the late Dr. Bayes (pseudo-homœopath), but he did her no good. Ordered her to leave off Manna, but made no other change in regimen.

*Diagnosis of remedy:*

Sadness before menses, *Am-carb.*, *Bell.*, *Berb.*, *Calc.*, *Caust.*, *Con.*, *Cyc.*, *Ignat.*, *Lac.-c.*, *Lac.-d.*, *Lycop.*, *Natr.-m.*, *Nitr-ac.*, *Phosph.*, *Puls.*, *Sep.*, *Stann.*, *Xanth.*

Fullness in rectum after stool, *Gymnocl.*, *Lycop.*

This reduces the number to *Lycop.*, which also has weakness after stool, urging to evacuate after stool, desire for solitude, fullness after eating, and chilblains. *Lycop.*$^{cm}$ (F. C.), one dose.

May 7th.—Feels much better in herself, much less constipation, no bleeding or fissure, only a bruised feeling in rectum; no feeling of swelling in rectum and less exhaustion after stool. Menses appeared on May 2d, lasting four days, more profuse; less previous depression. Fullness after eating much better.

May 14th.—Very much better. No fullness after meals. No swollen feeling in rectum, and less pain there. A little fissure and bleeding.

August 8th.—Has been quite well for two months, in spite of hard work.

August 25th.—Has visited Ventnor (by the sea), and has been rather constipated; the fissure has returned and has not yet healed, though it is not nearly so bad as formerly. No depression now before menses. Lycop.$^{mm}$ (Boericke), one dose.

September 28th.—Had a return of the depression from September 5th to 11th, but the menses then returned exact to time, lasting four days, natural in color, and without any pain. Fissure has not returned lately.

November 3d.—Last menses natural, only a little melancholy the first morning.

November 19th.—Slight fissure in anus; constipated. Feet and hands cold, also nose, which sometimes looks blue-red like a chilblain. Lycop.$^{mm}$ (Boericke) daily for seven days.

March 15th, 1881.—Says she has never been so free from chilblains and fissures before. Menses returned last week, but without any of the former concomitants.

March 24th.—For some deafness and other catarrhal symptoms, with return of chilblains on hands, I gave her Sulphur$^{dm}$ (F. C.) daily for seven days.

April 11th.—Says she has been more free from chilblains and cracks in hands than for the last ten years.

May 9th.—A slight return of anal fissure last week before menses; has had no return of menstrual pain, as she used to have, since she has been under my treatment. Has now a bruised feeling in chest on stooping. The *Encyclopœdia* gives under *Phosph.* (symptom 2545), "Bruised pain in upper part of chest, on motion, stooping, and touch." This is Hahnemann's own symptom, nevertheless, in the *Index*, page 248, it is only registered as "Upper chest, bruised on motion." *Phosph.*$^{cm}$ (F. C.) daily for a week.

February 27th, 1882.—Reports no return of fissure; and the same good report on September 4th. On this latter day she complained of a stye in left lower lid, close to inner canthus, with shooting pain. *Baryta.-carb.*$^{cm}$ (Swan) cured it at once.

January 9th, 1884.—Reports that last July left side of face swelled from a decayed wisdom-tooth, which was then removed by a dentist. Now for three days left side of face has been swelled, a red shining swelling, with throbbing and burning pain. Gave Bellad.$^{20m}$ (F. C.) every three hours till better.

January 12th.—Reports that she improved after the first dose; the redness and shining ceased the same day; the swelling did not break and has gone, and the pain also.

There has been no return of the fissure to this day.

